# Androgen Receptor (AR)-TLR4 Crosstalk Mediates Gender Disparities in Hepatocellular Carcinoma Incidence and Progression

**DOI:** 10.7150/jca.30682

**Published:** 2020-01-01

**Authors:** Qiuju Han, Dan Yang, Chunlai Yin, Jian Zhang

**Affiliations:** Institute of Immunopharmaceutical Sciences, School of Pharmaceutical Sciences, Shandong University, Jinan 250012, Shandong, China

**Keywords:** AR, Gender bias, TLR4, Hepatocellular Carcinoma

## Abstract

**Background:** Androgen receptor (AR) has a role in regulating malignancies and gender disparities in hepatocellular carcinoma (HCC). Recently, TLR4 activation is demonstrated to be required for HCC progression; however, whether and how TLR4 interacts with AR is largely unknown.

**Methods:** The tumorigenesis was detected in female and male mice induced by DEN/CCL_4,_ then TLR4 and AR signals were detected in liver tissues by qPCR and FACS. The proliferation, colony formation and migration of HCC cell treated with TLR4 agonist LPS, or/and androgen DHT were evaluated* in vitro*. Furthermore, the expression of TLR4 and AR was detected by IHC in tissue microarray of HCC, and correlation of AR and TLR4 was defined.

**Results:** Male mice are more susceptible to develop HCC than female mice. Meanwhile, we found baseline TLR4 levels were higher in male mice than in female mice. AR expression in male mice was increased by treatment with DEN/CCL_4_. And, AR was constitutively expressed in human HCC cell lines. Dihydrotestosterone (DHT) stimulated TLR4 expression in both HepG2 and HepG2 2.15 cells, which could be blocked by silencing AR. On the other hand, treatment with LPS stimulated AR expression, but it was blocked by treatment with TLR4 antagonist and in cells deficient for TLR4. DHT treatment exacerbated TLR4-induced cellular proliferation, colony formation, migration, and invasion of HepG2 cells. The positive relationship between AR and TLR4 was confirmed in human HCC samples.

**Conclusions:** DHT-AR-TLR4 signaling enhances the development of HCC cells and facilitates their migration and invasion, demonstrating a mechanism underlying gender disparity in HCC.

## Introduction

Hepatocellular carcinoma (HCC) is a leading cause of cancer-related death, and is notably more prevalent in men than in women, with a male-to-female ratio ranging from 2:1 to 8:1 1-3. Many risk factors contribute to gender disparity in HCC incidence, including gender-specific lifestyle differences, alcohol consumption, smoking habits, and infection with hepatitis B virus (HBV) or hepatitis C virus (HCV) 4, 5.

The androgen receptor (AR) is a member of the nuclear steroid receptor superfamily 6, 7, and is a transcription factor activated by ligand-dependent and ligand-independent mechanisms 8. In response to stimulation by androgens, AR translocates into the nucleus, where it binds to androgen response elements (AREs), and regulates expression of genes related to cell growth and survival; it is in this role that AR serves an important regulator of pathogenesis in hepatocarcinogenesis 4. AR has also been shown to promote expression of HBV viral RNAs 9. Furthermore, both higher serum androgen concentrations and the presence of AR gene variants containing shorter CAG repeats (leading to higher AR activity) have been linked to increased risk of hepatocarcinogenesis, especially of HBV-mediated HCC 7, 10. In addition, androgen/AR signals promote cancer cell stemness through direct activation of Nanog homeobox (NANOG) transcription in HCC 10. These data suggest that androgen-AR signals may play key roles in promoting the development of HCC. However, clinical studies using anti-androgens (e.g. flutamide and cyproterone acetate) yielded disappointing results, and demonstrated no apparent clinical benefits for patients 11, 12. The association between AR and gender disparity in HCC initiation and progression has been well documented, but the detailed mechanisms underlying how AR regulates HCC remain to be completely elucidated 2, 13.

The role of the gut-liver axis in the development of liver diseases, including non-alcoholic steatohepatitis and HCC, has recently attracted a great deal of attention 14, 15. Pattern recognition receptors (PRR), including Toll-like receptors (TLRs), recognize gut-derived bacterial components, and stimulate the innate immune system in response. TLR4 has been demonstrated to play an important role in the promotion of HCC promotion, but not in the initiation of HCC, and it mediates increased cellular proliferation, expression of the hepatomitogen epiregulin, and prevention of apoptosis 14, 16, 17. TLR4 signaling promotes the development of HCC development in HCC induced by N′-N′-diethylnitrosamine (DEN) by increasing production of proinflammatory and cancer-related biomolecules, including cytokines, NANOG, Caspase-1, Ephrin-A1, NO, and B-cell CLL/lymphoma 6 (BCL6) 18, 19. Furthermore, TLR4 signaling plays a role in the migration and invasion of HCC cells. We reported that TLR4 signaling contributes to a COX2/PGE2/STAT3 positive feedback loop in HCC cells 20. In addition, the TLR4 rs1927914 polymorphism is associated with increased risk of HCC recurrence 21. Taken together, these data suggest a critical role for TLR4 in the promotion of HCC.

Based on the importance of TLR4 and AR in HCC development, we wanted to know whether and how TLR4 crosstalk occurs with sex hormone receptors, especially AR, during the development of HCC. Here, we report that the baseline expression of TLR4 was higher in male mice than in female mice. This gender disparity was further increased by treatment with DEN/CCL4*.* TLR4 showed a positive relationship with AR expression in human HCC tissues. Further investigation demonstrated that DHT-AR is critical for TLR4 signal-mediated promotion of HCC, suggesting that TLR4 is essential for gender disparities observed in HCC. These findings provide new insight for improving the efficacy of HCC treatment in the clinic.

## Material and Methods

### Mouse model of DEN-induced HCC

A mouse model of DEN-induced HCC was generated (described in Supplementary Methods). 6-week-old C57BL/6 mice were obtained from HFK Bioscience Co, Ltd (Beijing, China). Beginning at fourteen days of age, female and male mice (5 mice per group) received weekly intraperitoneal *(i.p*.) injections of 20 mg/kg DEN for three weeks (Sigma, USA), followed by weekly *i.p.* injections of 100 μl CCL4 dissolved in olive oil for six weeks. The procedure was divided into three stages; early (7-21 weeks), middle (22-42 weeks), and late (43 week-sacrificial endpoint), as previously described 16. Evaluation of tumor number and size was determined as described by counting the number of visible tumors and measuring the size of the largest tumor with calipers, the rate of tumor incidence was is recorded 16. All animals were kept in standard laboratory conditions and provided with food and water ad libitum. All animal experiments were approved by the Ethics Committee of Shandong University.

### Cell lines and reagents

The HCC cell lines HepG2, H7402, Hepa1-6, and HepG2.2.15 were cultured and maintained in our laboratory. All cell lines were grown in DMEM (Gibco, USA), containing 1% penicillin-streptomycin and supplemented with 10% fetal bovine serum (FBS). Cell cultures were incubated at 37°C in 5% CO_2_. LPS isolated from *Escherichia coli* (0111:B4), natural AR ligand dihydrotestosterone (DHT; T1500), and estrogen (E2) (E2758) were purchased from Sigma-Aldrich (St. Louis, MO). The TLR4 signaling inhibitor, TAK-242, was obtained from Invivogen (San Diego, CA, USA).

### Quantitative real-time PCR analysis

1.5×10^5^ HepG2 or Hep1-6 cells/well were plated into 12-well plates, and were treated with DHT or LPS. Total RNA from cells and liver tissues was extracted using the Trizol reagent (Invitrogen, Carlsbad, CA, USA), and was used to generated cDNA using Moloney Murine Leukemia Virus Reverse Transcriptase (M-MLV; Invitrogen) according to the manufacturer's protocol. cDNA amplification was performed using real-time PCR with FastStart Universal SYBR Green Master (Roche, Switzerland) on an iCycleriQ real-time PCR system (Bio-Rad, Hercules, CA, USA). GAPDH and β-actin genes were used to normalize gene expression. The primers used in this study are described in Table 1.

### Immunofluorescence microscopy

Immunofluorescence was performed using primary antibodies to AR (Abcam Cambridge, UK), and DyLight^TM^ 549-conjugated goat anti-rabbit secondary antibody (Abbkine, Wuhan, China). Quantification was performed using Image J and Photoshop software, analyzing at least 10 images per mouse for AR staining.

### Cell proliferation assay

The MTT cell proliferation reagent (Roche Molecular Biochemical, Mannheim, Germany) was used to evaluate cell growth, according to the manufacturer's instructions. Briefly, 5×10^3^ cells/well were plated into 96-well plates. After sample preparation, 20 µL of MTT reagent was added, and cells were incubated at 37 °C for another 3h. Absorbance at 570 nm was recorded by a scanning multi-well spectrophotometer (Bio-Rad, Hercules, CA).

### Western blot analysis

Cells or liver tissue was homogenized using lysis buffer with containing a protease inhibitor cocktail (Beyotime Biotechnology, Shanghai, China). Total protein (30 µg) from each sample was separated on a 10% SDS-polyacrylamide gel, and then transferred onto PVDF membranes. Membranes were incubated for 1 h in blocking buffer containing 5% milk, then incubated with anti-β-actin (Santa Cruz Biotechnology, CA), anti-AR (Abcam, CA), or rabbit anti-TLR4 (PL Laboratories, British Columbia), diluted 1:2000 overnight at 4 °C. After overnight incubation, membranes were incubated with peroxidase conjugated goat anti-rabbit IgG (Santa Cruz Biotechnology, CA) for 1 h at room temperature. Proteins were visualized using Immobilon Western Chemiluminescent HRP Substrate (Millipore, Billerica, MA) and detected with Alpha Ease FC software (Bio-Rad, Hercules, CA) 22.

### Immunohistochemical Staining (IHC)

A human hepatoma tissue microarray (OD-CT-DgLiv02 and HLiv-HCC060CD-01) was obtained from Outdo Biotech (Shanghai, China). Paraffin-embedded and formalin-fixed mouse liver samples were cut into 5 μm sections, which were then processed for immunohistochemistry as described 23. After incubation with an antibody against TLR4 (Santa Cruz Biotechnology) or AR (Abcam), samples were kept overnight at 4 °C. IHC staining was evaluated using Image-Pro Plus v6.2 software. The relative expression level of each protein was quantified according to integrated optical density.

### Analysis of apoptosis by FACS

1.5×10^5^ cells/well were plated into 12-well plates. Cells were treated with DHT or LPS. Twenty-four hours later, cells were washed, harvested, and re-centrifuged in 200 μl 1× annexin-binding buffer. Cells were incubated with 5 μl Alexa Fluor® 488 Annexin V and 2.5 μl of propidium iodide (PI) (Bestbio, Shanghai, China) at room temperature for 15 min, then analyzed by flow cytometry (FACSCalibur, BD).

### Transwell migration assays

The migration assay was performed using 24-well transwell inserts with a pore size of 8 μm (Corning, USA). Initially, HepG2 cells treated with DHT or LPS were seeded into the upper chamber in serum-free medium. DMEM with 10% FBS was added to the lower chamber, as a chemoattractant. After 6 h incubation at 37 °C, the cells that traversed the membrane were fixed in methanol and stained with 0.1% crystal violet. For quantification, the number of migrated cells was calculated by counting at least five random separate fields.

### Colony formation assay

For the clonogenic colony formation assay, 500 cells/well were seeded in 6-well plates. After 14 days, visible colonies were observed using the naked eye, fixed with 4% formaldehyde, and stained with 0.1% crystal violet. Colonies with a diameter greater than 1 mm were counted.

### Statistical analysis

Statistical analysis was performed using paired Student's *t*-test. Statistical significance was determined as ** *P* < 0.01 and * *P* < 0.05 compared with control.

## Results

### Male mice exhibit increased susceptibility to HCC development

**Figure 1:**
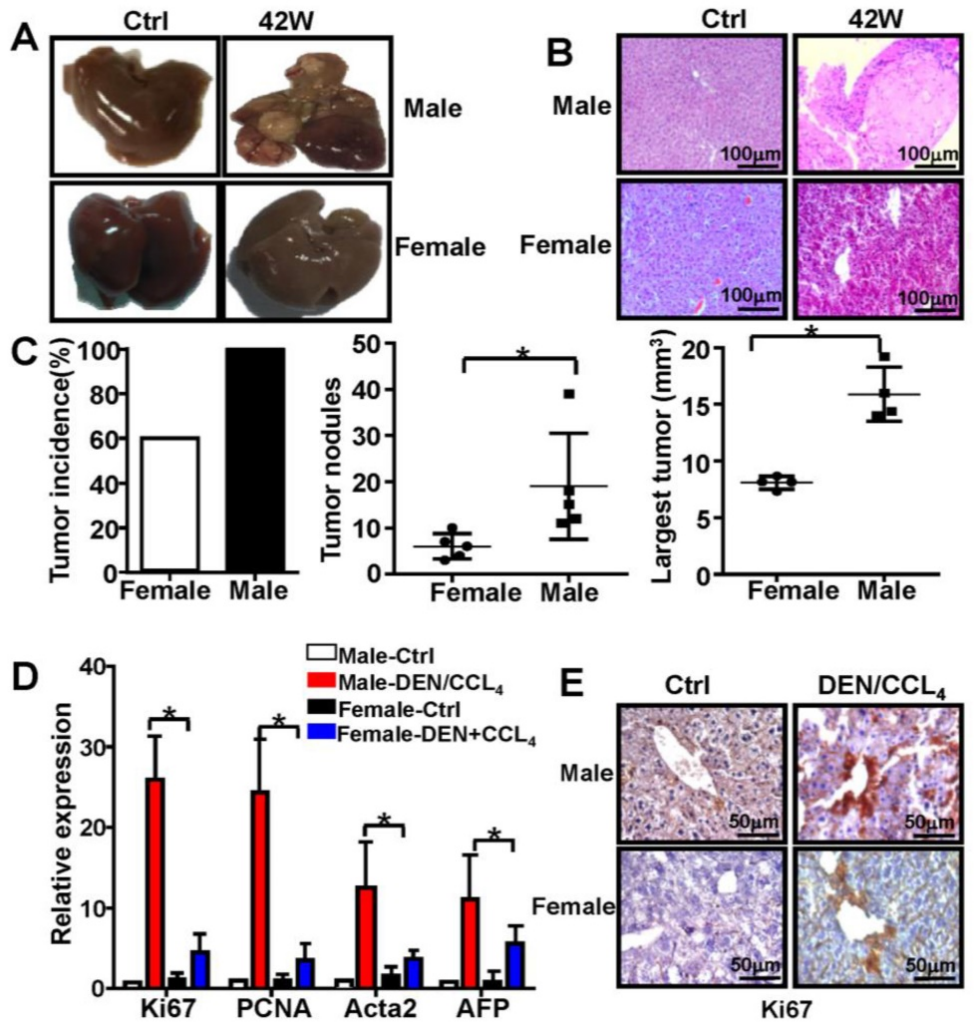
** Male mice are more susceptible to develop HCC than female mice.** C57BL/6 mice were injected three times with DEN (100 mg/kg i.p.) starting at the age of 2 weeks, followed by six injections of CCl4 (0.5 ml/kg i.p.); mice were sacrificed different time points after DEN treatment (×200). B&C**.** Male and female mice were sacrificed 42 weeks after DEN injection. The appearance of liver tissue **(A)** and H&E staining **(B)** from DEN-induced mice were shown. **(C)** Tumor incidence, tumor number, and largest tumor size were assessed 42-weeks after DEN injection in female and male mice. **(D)** The proliferation marker Ki67, PCNA, and clinical indicators of HCC, AFP and Acta2, were analyzed by q-PCR. Data are represented as mean ± SEM (n=5). **(E)** Proliferation of hepatocytes indicated by Ki67 protein expression was analyzed by IHC (×400). * P < 0.05: Male-DEN/CCL4 versus Female-DEN/CCL4.

The incidence and mortality of liver cancer incidence is significantly higher in male mice than in female mice 1, 24. In the present study, male and female mice were subjected to the combination of treatment with DEN and CCl_4_ (**Figure S1**). The tumors induced by this treatment demonstrated typical features of HCC in male mice (**Figure 1A & 1B**), and the spleens of male and female mice did not show any significant differences (**data not shown**). Moreover, compared to female mice, there was a profound increase in tumor number and size in male mice 44 weeks after the initial DEN/CCl_4_ treatment (n=5) (**Figure 1C**). qPCR analysis revealed that expression of Ki67, proliferating cell nuclear antigen (PCNA), Acta2, and alpha-fetoprotein (AFP) was markedly increased in male mice treated with DEN/CCl_4_, and displayed expression profiles characteristic of HCC (n=5) (**Figure 1D**). By IHC analysis, Ki67 protein expression was higher in the liver tissue from male mice treated with DEN/CCl_4_ than in female mice (**Figure 1E**). These data confirm that there is a gender disparity associated with the development of HCC.

### Both TLR4 and AR expression is increased in this model of male HCC

**Figure 2:**
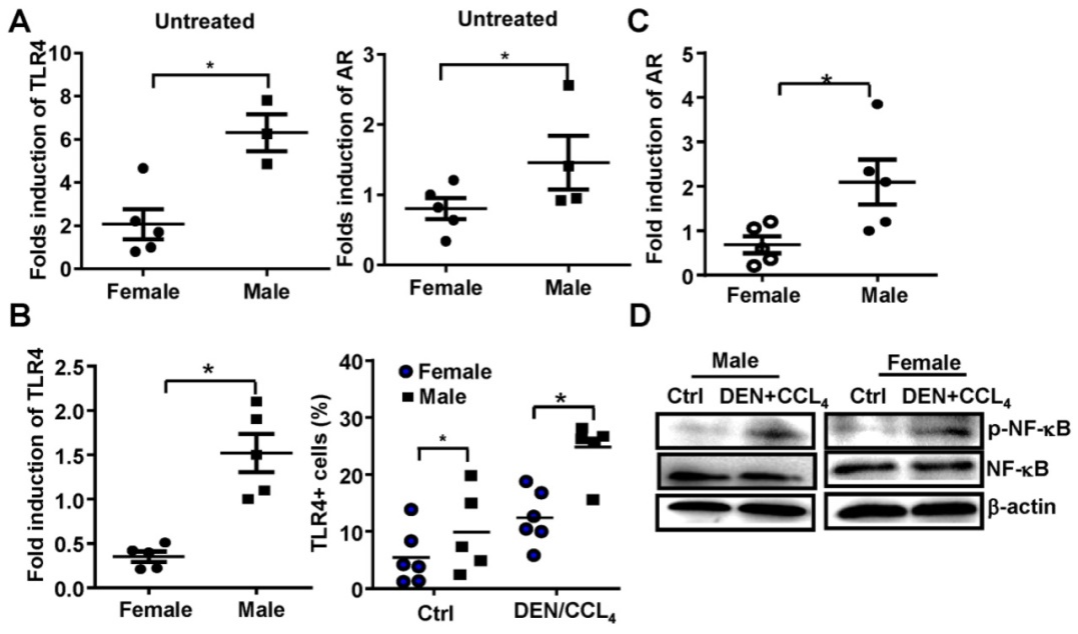
** The expression of both TLR4 and AR is increased in a male HCC model. (A)** TLR4 and AR expression were evaluated by qPCR in untreated-female and male mice. **(B)** The mRNA of TLR4 and TLR4-positive hepatocytes from female and male mice treated with DEN were evaluated by qPCR and FACS. **(C)** AR levels were measured in DEN treated female and male mice by qRT-PCR. **(D)** NF-κB activation in hepatocytes from female and male mice was detected by Western Blot. Data are represented as mean ± SEM, (n=5). Bar graph shows quantification results.

Previous reports demonstrate that AR promotes gender disparities in cancer 10, and TLR4 plays an important role in promoting liver cancer 16. Therefore, we evaluated whether AR was associated with TLR4 signaling at the initiation and promotion stages of HCC in this model. We initially found that the baseline mRNA levels of TLR4 and AR were significantly higher in male mice than that in female mice (**Figure 2A**). Following DEN/CCL_4_ injection, we observed TLR4 positive hepatocytes were increased in liver tissue of male mice (n=5) (**Figure 2B**); this was accompanied by an elevation of AR mRNA (**Figure 2C**). Furthermore, NF-κB activation in liver tissue from male mice were confirmed by western blot assays (**Figure 2D**)**.** These findings indicate there may be a relationship between TLR4 and AR in HCC development.

### AR and TLR4 positively regulate each other

**Figure 3:**
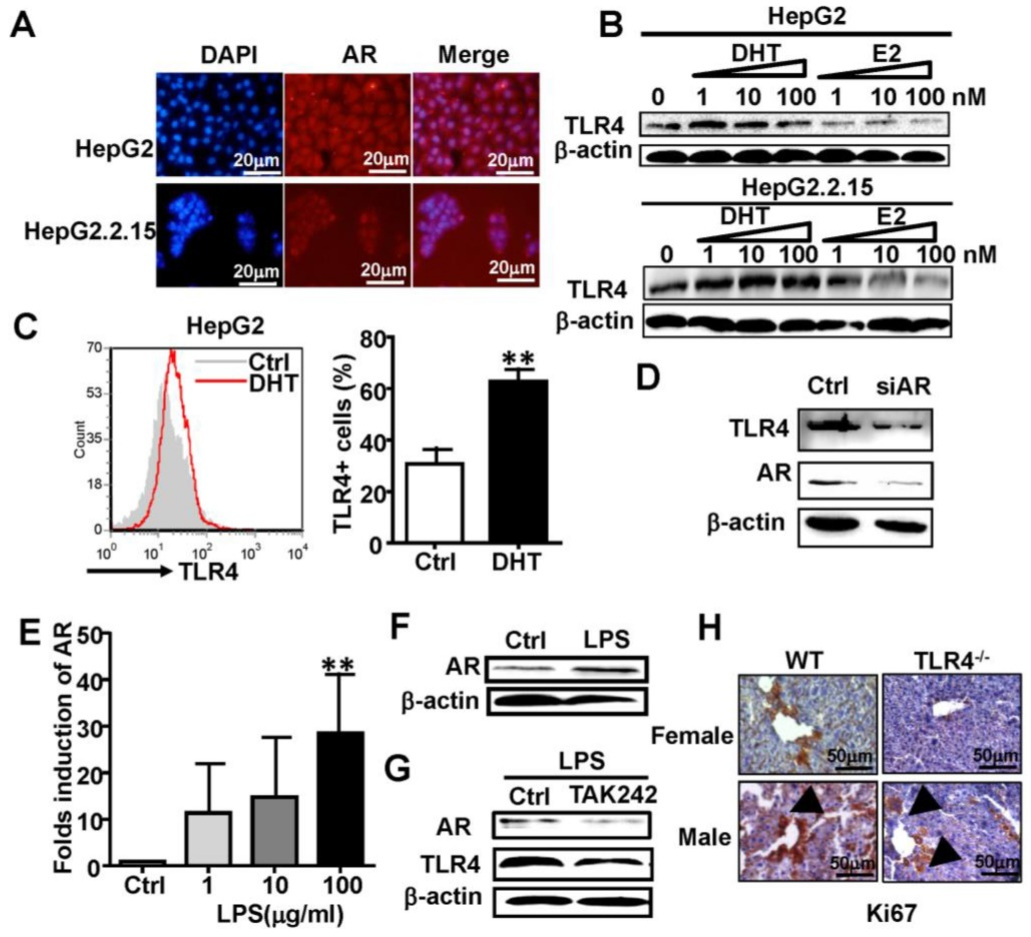
** TLR4 and AR up-regulate each other. (A)** AR expression was analyzed by immunofluorescent staining in HepG2 and HepG2.2.15 cells; nuclear stain indicated by DAPI stain. The MERGE is the merge of DyLight 549 and DAPI (×400). **(B)** Western blot analysis showing the protein expression of TLR4 in HepG2 and HepG2.2.15 cells treated by different concentrations of DHT or E2 for 48 h. **(C)** TLR4 assay by FACS for HepG2 cells was performed following treatment with control or 100 nM DHT for 24h. Bar graph shows quantification results; data are represented as mean ± SEM. **(D)** Western blot analysis showing the protein expression of TLR4 and AR in HepG2 cells transfected with siRNA targeting AR (siAR), or scramble siRNA (Ctrl), for 48h. AR levels were measured in LPS treated HepG2 cells by qRT-PCR **(E)** and western blot **(F)**.** (G)** TLR4 and AR protein expression levels were assayed by western blot in HepG2 cells treated with the TLR4 inhibitor TAK242. **(H)** Ki67 protein expression was analyzed by IHC at 23 weeks after DEN treatment in wild type (WT) and TLR4^-/-^ male and female mice (×400).

To clarify the relationship between AR and TLR4 signaling in HCC, we studied the human HCC cell lines HepG2 and HepG2.2.15, which constitutively express AR at a relatively high level (**Figure S2**)**.** Immunofluorescence staining confirmed the expression of AR in HepG2 and HepG2.2.15 cells (**Figure 3A**). HepG2 and HepG2.2.15 cells were treated with androgen DHT or estrogen (E2) for 48 h. Treatment with DHT elevated TLR4 the expression of TLR4, while E2 treatment down-regulated TLR4 in HepG2 cells (**Figure 3B, top**). A similar trend in TLR4 expression changes was observed in HepG2.2.15 cells (**Figure 3B, bottom**). Meanwhile, FACS assay revealed the percentage of TLR4 positive HepG2 cells was increased by DHT treatment (**Figure 3C**). However, silencing of AR by siRNA abolished the DHT-induced up-regulation of TLR4 in HepG2 cells (**Figure 3D**). We stimulated HepG2 cells with the TLR4 agonist, LPS, for 24 h; the expression of AR at both the mRNA and protein levels was increased (**Figure 3E-F**); this increase in AR was abrogated by the TLR4 inhibitor TAK242 (**Figure 3G**). In addition, the level of Ki67 in WT male mice treated with DEN/CCL4 was higher than that in WT female mice; this was profoundly decreased in TLR4^-/-^ mice (**Figure 3H**). These results suggest that the positive regulation between AR and TLR4 signaling is critical for HCC initiation and development.

### AR enhances TLR4-induced tumor characteristics in HCC cells

**Figure 4:**
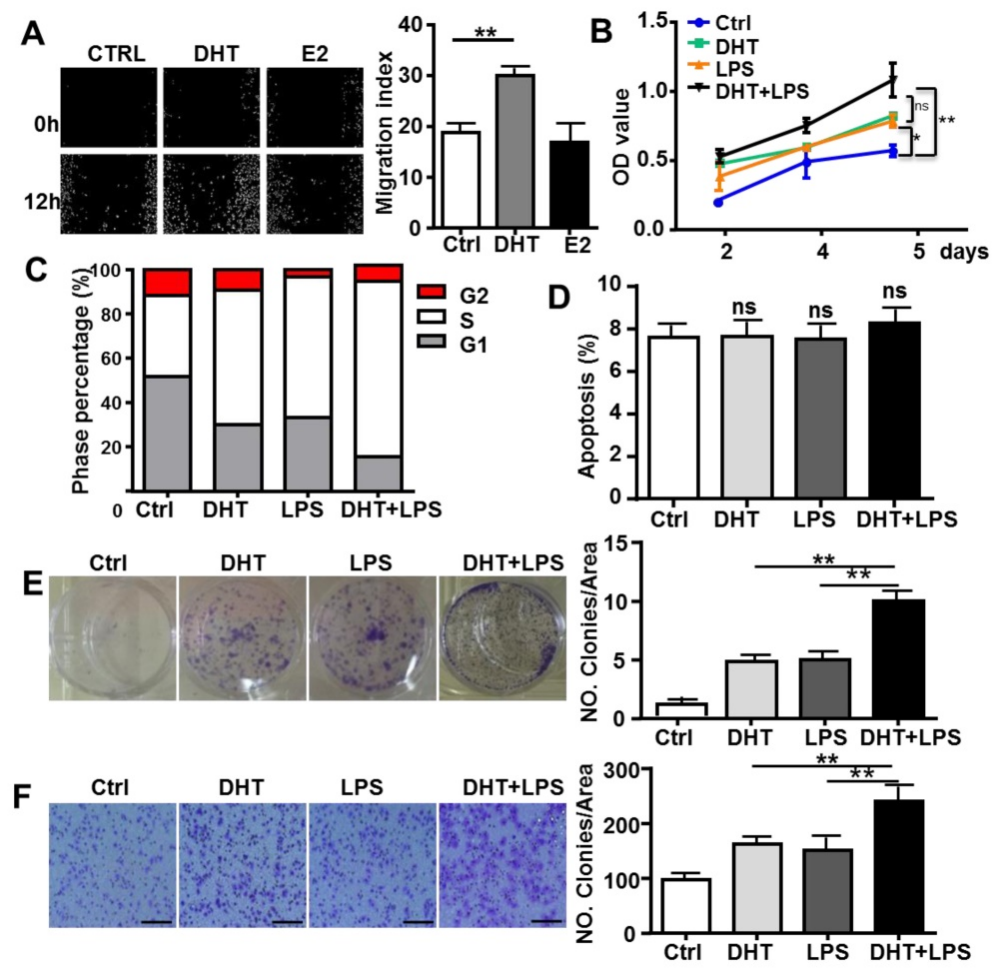
** AR enhances TLR4-induced HCC proliferation, colony formation, and migration. (A)** HepG2 cells were treated with DHT (100 nM) or E2 (100 nM) for 12 h. The migration rate was detected by Image-Pro Plus. Bar graph shows quantification results. Data are represented as mean ± SEM. **(B)** AR positive-HepG2 cells were treated with LPS (10 µg/ml), DHT (100 nM), or with a combination for 2, 4, and 5 days, and the proliferation rate was assessed by the MTT assay. **(C)** HepG2 cells were incubated with LPS (10 µg/ml) and DHT (100 nM) for 48 h, and cell cycle distribution of was analyzed by FACS. **(D)** HepG2 cells were treated with DHT, LPS, or with the combination indicated for 24 h, then Annexin V/PI staining was employed to detect the apoptosis of cells. **(E)** HepG2 cells were treated with LPS (10 µg/ml), DHT (100 nM), or with a combination for 6 h. After 14 days, colony formation was detected by Crystal Violet stain. **(F)** HepG2 cells were treated LPS (10 µg/ml) and DHT (100 nM) for 6 h. The migration rate was evaluated by Crystal Violet staining (the scale bar is 100nm). Bar graph shows quantification results. Data are represented as mean ±SEM.

**Figure 5:**
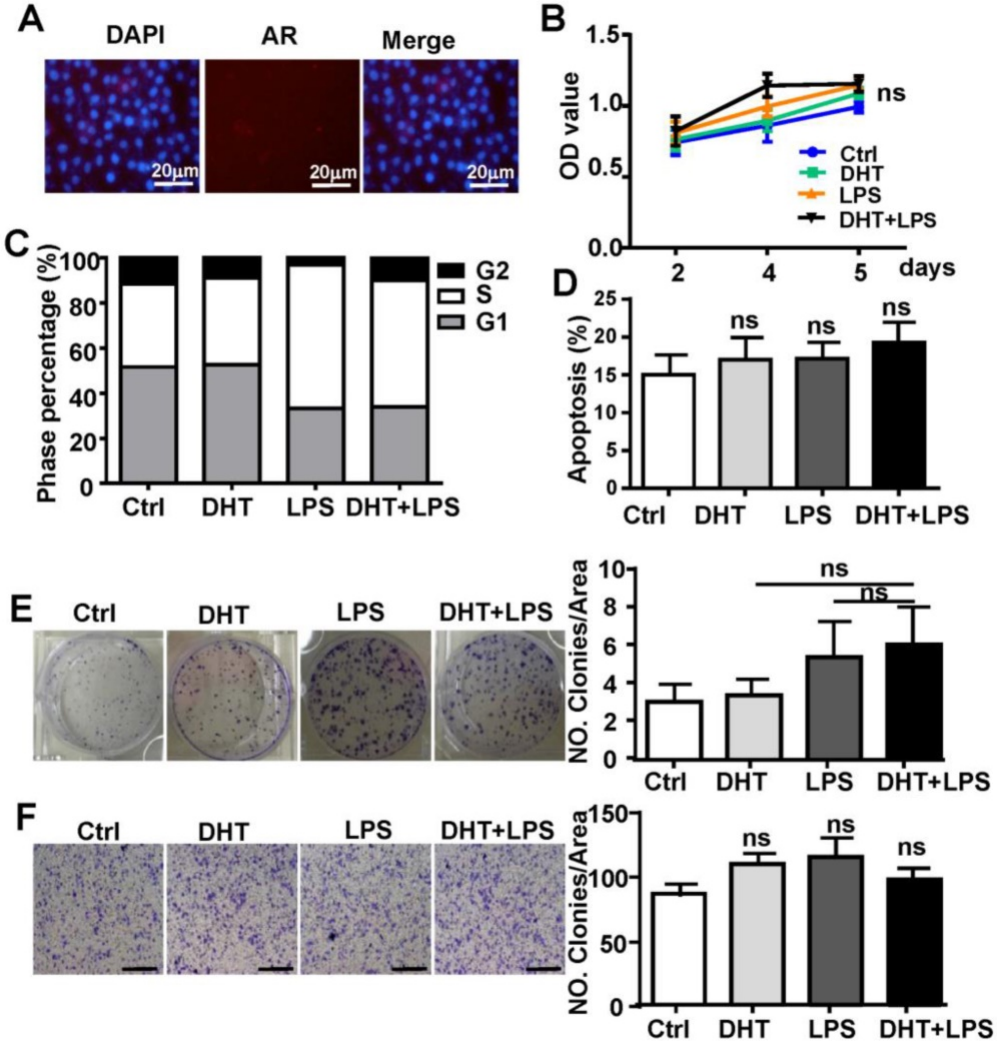
** DHT has no effect on the growth of Hepa1-6 cells. (A)** AR expression was analyzed by immunofluorescent staining in Hepa1-6 cells; nuclear staining is indicated by DAPI stain. The MERGE is the merge of DyLight 549 and DAPI (×400). **(B)** Hepa1-6 cells were treated with LPS (10 µg/ml), DHT (100 nM), or with a combination for 2, 4, and 5 days, and the proliferation rate was assessed by the MTT assay. **(C)** Hepa1-6 cells were incubated with LPS (10 µg/ml) and DHT (100 nM) for 48 h, then the cell cycle distribution was analyzed by FACS. **(D)** Hepa1-6 cells treated with DHT, LPS, or with the combination indicated for 24 h, then Annexin V/PI staining was employed to detect apoptosis of the cells. **(E)** Hepa1-6 cells were treated with LPS (10 µg/ml), DHT (100 nM), or with a combination for 6 h. After 14 days, colony formation was assessed by Crystal Violet stain (×200). **(F)** Hepa1-6 cells were treated with LPS (10 µg/ml) and DHT (100 nM) for 6 h. The migration rate was evaluated by Crystal Violet staining (the scale bar is 100nm). Bar graph shows quantification results. Data are represented as mean ± SEM.

To determine if AR-associated signaling facilitates TLR4-induced HCC development, HepG2 cells were treated with 100 nM DHT or 100 nM E2. After stimulation for 6 h, we observed that DHT promoted the wound healing capacity of HCC cells, while E2 did not show significant effects (**Figure 4A**). Compared to control, DHT- and LPS-treated cells, the proliferation of HCC cells was augmented by treatment with a combination of LPS and DHT (LPS+DHT) (**Figure 4B**); simultaneously, the proportion of HepG2 cells in S-phase was increased (**Figure 4C**), but the apoptosis of HepG2 cells was not affected by DHT+LPS (**Figure 4D**). Furthermore, colony formation assays revealed that both DHT and LPS stimulated colony growth (**Figure 4F**) and enhanced the migratory ability (**Figure 4G**) of HepG2 cells, which was further enhanced by treatment with a combination of LPS and DHT. However, these phenomena were not observed in Hepa1-6 cells, which exhibited low levels of AR (**Figure 5**). Therefore, these data suggested that AR signaling exacerbates TLR4-induced proliferation, colony formation, and migration of HCC cells.

### Positive relationship between TLR4 and AR expression in human HCC tissues

**Figure 6:**
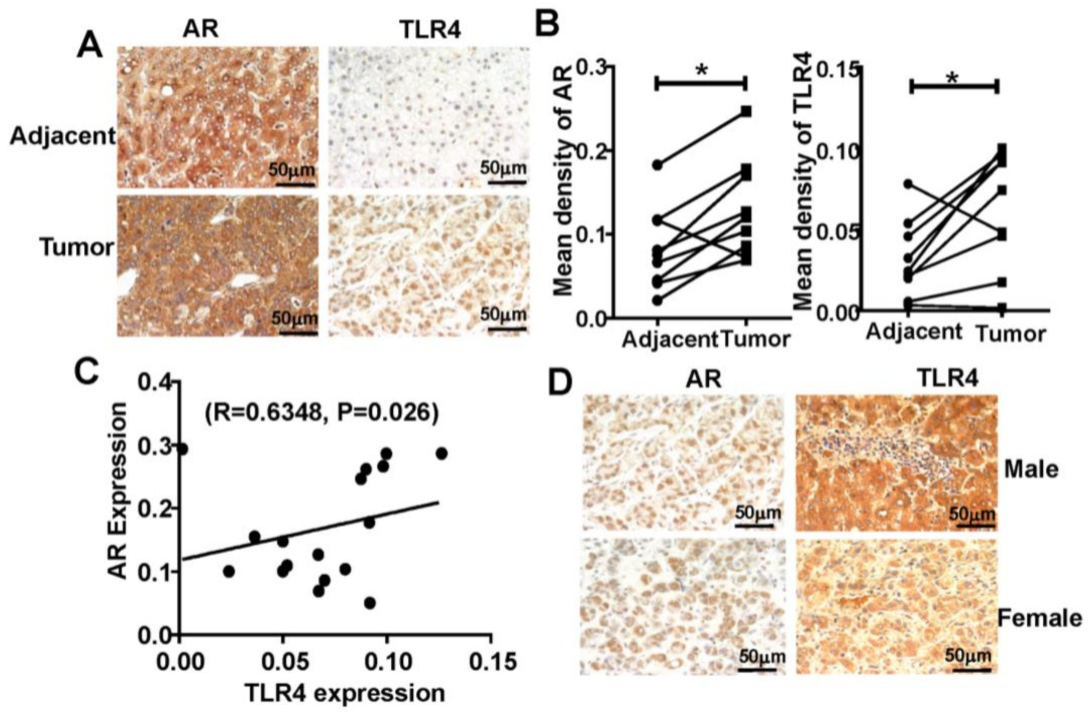
** Positive correlation of AR and TLR4 in HCC patients. (A)** IHC analysis of TLR4 and AR expression in 30 paired HCC tissues and their corresponding adjacent normal tissue (×400). **(B)** IHC staining was evaluated using Image-Pro Plus v6.2 software, and the relative expression level of each protein was quantified based on integrated optical density. Statistical significance of TLR4 and AR between HCC and adjacent tissues was indicated at *P* < 0.05. **(C)** Correlation between the relative protein levels of AR and TLR4 in 30 HCC tissue specimens. **(D)** IHC analysis of TLR4 and AR expression in male and female HCC tissue microarray (×400).

To extend our findings in mice to human HCC, we analyzed TLR4 and AR expression using tissue microarray, containing matched pairs of tumor and peritumoral liver tissue from 22 male and 8 female patients with primary HCC. We found that TLR4 and AR were expressed more highly in HCC tumor tissue than in adjacent liver tissue (**Figure 6A-B**), and we found that the expression of TLR4 was positively correlated with AR in HCC tumors (R=0.6348, P=0.026; **Figure 6C**). Furthermore, we found that the expression of AR in male is higher than that in female HCC. And the similar trend was observed for detection of TLR4 (**Figure 6D**).These results support the positive relationship between TLR4 and AR in HCC development.

## Discussion

Here, we show that TLR4 expression was increased in male mice treated with DEN/CCL4*.* We discovered that a positive feedback loop involving DHT-AR and TLR4 signaling is critical for the initiation and development of HCC. We conclude that DHT-AR-TLR4 signaling mediates promotion of HCC, which is a critical aspect of the gender disparity in HCC.

TLR4 promotes HCC via several mechanisms, including increasing expression of NANOG, Caspase-1, Ephrin-A1, NO, and BCL6 and increasing the number of T regulatory lymphocytes 17, 18, 25. TLR4 also facilitates the proliferation of HCC cells, mediated by a COX-2/PGE2/STAT3 positive feedback loop, and induces the production of pro-inflammatory and malignancy related molecules 20. We propose that TLR4 signaling also contributes to the gender disparity observed in HCC. Our data demonstrate that TLR4 levels are increased in DEN/CCL_4_-treated male mice, that and DHT-AR and TLR4 signaling are involved in a positive feedback loop (**Figure 2 and Figure 3**)**.** In addition, in line with our findings, estrogen restricts TLR4 and TNF-α, but not TLR3 and NLRP3 responses, contributing to a proinflammatory hepatic microenvironment in males, which imparts increased risk to men with chronic viral hepatitis to progress to cirrhosis or HCC 26.

Previous studies found that AR activation could convene more than 200 kinds of regulatory molecules, including transcriptional factors, kinases, molecular chaperones, cytoskeleton proteins, and molecules related to histone modification. Importantly, PGE and STAT3 are among them, and we preciously found that TLR4 signaling could promote the proliferation and induce multidrug resistance of HCC cells to chemotherapeutics, in which COX-2/PGE2/STAT3 positive feedback loop 20, 27, 28. In this study, although we found AR and TLR4 positively regulate each other (**Figure 3**), the exact molecular mechanism has not been enucleated, we still need further study to explore it.

Our study demonstrates that AR can promote HCC tumorigenicity; this is supported by evidence that mice lacking hepatic AR had less hepatocarcinogenesis after exposure to a higher dose of DEN 4, 29. Treatment with the chemotherapeutic agent cisplatin suppresses AR expression by two distinct mechanisms: by increasing miR-34a-5p to suppress AR expression and by altering AR ubiquitination to accelerate AR protein degradation; suppressed AR expression may result in up-regulation of ULBP2, a natural-killer group 2 member D (NKG2D) ligand, to enhance the cytotoxicity of NK cells against HCC 30. Another study reported that LPS-induced apoptosis in HepG2 cells stably expressing shAR, but not in control HepG2 cells 31, suggesting that AR may be involved in the apoptotic response of hepatocytes. Our data is not consistent with this observation, as we observed no effect of DHT treatment on HCC cell apoptosis, while AR signaling exacerbated TLR4-induced HCC proliferation, colony formation, and migration (**Figure 4**).

Similar to the gender disparities seen in human HCC, gender differences are also observed in mouse models of HCC, including both genetically or chemically induced HCC. Sex hormones and inflammatory responses have been implicated in the gender disparity of HCC; such studies have often used a mouse model that develops HCC upon exposure to DEN. This model incorporates chronic injury, inflammation, fibrogenesis, and CCl4-mediated increases in endotoxin levels 16, 32. Thus, male mice are usually used to model HCC.

In addition, various immune cells in the tumor microenvironment influence the progression of HCC 3, 19. AR alters NK cells cytotoxicity by suppressing Interleukin (IL)-12A expression in HCC cells, revealing a relationship between the cytotoxicity of NK cells and AR in HCC 13, 33. Thus, the potential connection between AR and immune surveillance during HCC progression merits further evaluation. Although we found expression of TLR4 was positively correlated with TLR4 in tumors (**Figure 6**), the relationship of these molecules with survival time needs further clarification. Targeting AR with ASC-J9® or other small molecules may have potential to be developed to suppress HCC progression more effectively in near future 34.

## Conclusions

Our data suggest that DHT-AR-TLR4 signaling enhances the development of HCC cells and facilitates their migration and invasion. These findings suggest that AR and TLR4 are potential therapeutic targets in the treatment of HCC, although further mechanistic studies are warranted.

## Supplementary Material

Supplementary figures.

## Figures and Tables

**Table 1:** The primers used in this study

Gene name	Sequence 5' → 3'	Size (bp)
Human GAPDH	F:GAAGGTGAAGGTCGGAGT	155
	R:CATGGGTGGAATCATATTGGAA	
Human AR	F: GACGACCAGATGGCTGTCATT	106
	R: GGGCGAAGTAGAGCATCCT	
Human ERα	F: GGGAAGTATGGCTATGGAATCTG	220
	R: TGGCTGGACACATATAGTCGTT	
Human ERβ	F: AGCACGGCTCCATATACATACC	199
Mouse β-actin	F:AGAGGGAAATCGTGCGTGAC	128
	R:CAATAGTGATGACCTGGCCGT	
	R: CACCTCCATTGTCCCTGTTTTAT	
Mouse AFP	F: CTTCCCTCATCCTCCTGCTAC	145
	R: ACAAACTGGGTAAAGGTGATGG	
Mouse PCNA	F: TTTGAGGCACGCCTGATCC	135
	R: GGAGACGTGAGACGAGTCCAT	
Mouse Ki67	F: ACCGTGGAGTAGTTTATCTGGG	126
	R: TGTTTCCAGTCCGCTTACTTCT	
Mouse Acta2	F: GTCCCAGACATCAGGGAGTAA	102
	R: TCGGATACTTCAGCGTCAGGA	
Mouse AR	F: CTGGGAAGGGTCTACCCAC	128
	R: GGTGCTATGTTAGCGGCCTC	

## Abbreviations

AR: androgen receptor
HCC: Hepatocellular carcinoma
DEN: N′-N′-diethylnitrosamine
AFP: alpha-fetoprotein
PCNA: proliferating cell nuclear antigen
IL: Interleukin
TLR4: Toll like receptor 4
E2: Estradiol
DHT: Dihydrotestosterone
NANOG: Nanog homeobox
IHC: Immunohistochemical
BCL6: B-cell CLL/lymphoma 6
PRR: pattern recognition receptor
COX2: cyclooxygenase 2
