# Supplementary material for: Androgen Receptor (AR)-TLR4 Crosstalk Mediates Gender Disparities in Hepatocellular Carcinoma Incidence and Progression
Source: J Cancer. 2020 Jan 1;11(5):1094–103. doi: 10.7150/jca.30682 (PMC6959060; doi:10.7150/jca.30682)
Supplement: Supplementary file 1 — Supplementary figures. [file jcav11p1094s1.pdf]

Figure S1 Schematic diagram for the procedure of the mouse HCC model and assay

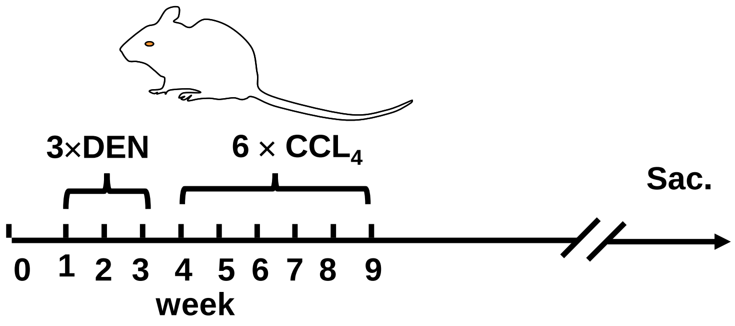

Figure S1. Schematic diagram for the procedure of the mouse HCC model and assay. C57BL/6 mice were injected three times with DEN (100 mg/kg, i.p.) at the age of 2 weeks followed by six injections of CCl_4 (0.5 ml/kg i.p.) and sacrificed at different time points after DEN treatment.

Figure S2. AR and ER expression levels in human HCC cell lines

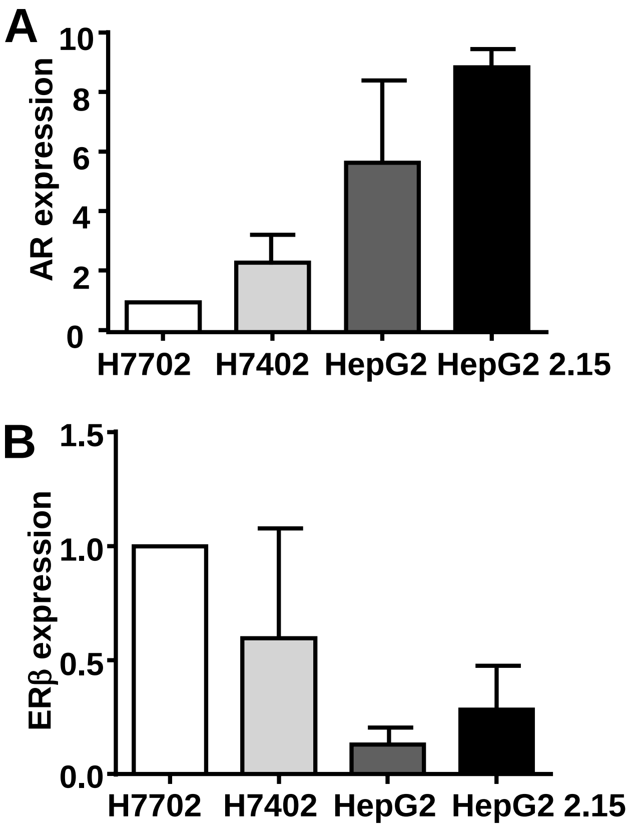

Figure S2. AR and ER are constitutively expressed in human HCC cell lines. The mRNA levels of AR (A) and ERβ (B) were detected in the indicated cell lines. Data are represented as mean ± SEM.
